# Physical Activity and Hepatocellular Carcinoma Outcomes: a Narrative Review of Pre-clinical, Observational, and Interventional Evidence

**DOI:** 10.1007/s12029-026-01420-2

**Published:** 2026-03-05

**Authors:** Nadia Kim, Lisa Alcock, Silvia Del Din, Helen L. Reeves, Samuel T. Orange

**Affiliations:** 1 National Institute for Health and Care Research (NIHR) Newcastle Biomedical Research Centre (BRC), Newcastle University, Newcastle upon Tyne, UK; 2 Translational and Clinical Research Institute, Faculty of Medical Sciences, Newcastle University, Newcastle upon Tyne, UK; 3 Newcastle University Centre for Cancer, Newcastle upon Tyne, UK; 4 School of Biomedical, Nutritional and Sport Sciences, Faculty of Medical Sciences, Newcastle University, Newcastle upon Tyne, UK; 5 Emles Bioventures, London, UK

**Keywords:** Hepatocellular carcinoma, Physical activity, Exercise, Frailty, Sarcopenia

## Abstract

**Purpose:**

Hepatocellular carcinoma (HCC) is a leading cause of cancer-related mortality worldwide, with incidence and mortality projected to rise. Most patients present with advanced or unresectable disease, where treatment efficacy is often constrained by frailty and impaired physical function. As physical function has emerged as a key determinant of treatment eligibility and survival, there is growing interest in physical activity as a supportive intervention in HCC care.

This narrative review synthesises observational, pre-clinical, and trial evidence on the role of physical activity in patients with HCC, examining its potential to improve treatment tolerance, quality of life, and survival.

**Methods:**

A literature search was conducted across four databases (PubMed, Scopus, Web of Science, Embase) using keywords related to HCC, physical activity, exercise, and survivorship.

**Results:**

Pre-clinical studies reveal multiple mechanisms through which physical activity may enhance anti-tumour responses, including metabolic reprogramming, modulation of oncogenic signalling and immune activation. Observational studies suggest that frailty and sarcopenia - markers of reduced physical function, are possibly associated with early treatment discontinuation and shorter survival in HCC. Preliminary interventional data suggest that structured exercise programmes, delivered in hospital, outpatient, telehealth, or mobile formats, can improve frailty, preserve muscle mass, and may potentially support survival. However, clinical studies to date are limited by small sample sizes, non-randomised designs, and short follow-up periods.

**Conclusion:**

Current evidence provides a biologically plausible and clinically promising rationale for integrating physical activity into HCC care pathways. While findings are encouraging, robust randomised trials are needed to establish efficacy, define optimal exercise regimens, and evaluate long-term outcomes.

## Introduction

The global burden of liver cancer remains substantial, with close to 1 million cases per year [1]. Hepatocellular carcinoma (HCC), which accounts for approximately ~ 75–85% of primary liver cancers, is a leading cause of cancer-related mortality worldwide, responsible for around 830,000 deaths annually [2]. In the UK, HCC is the 18th most common cancer overall; it is characterised by a poor prognosis, with a five-year survival rate of just 13% [3].

Most patients with HCC present with unresectable disease and rely on palliative therapies, including locoregional treatments such as transarterial chemoembolisation (TACE) and systemic therapies like tyrosine kinase inhibitors and immune checkpoint inhibitors [6]. These treatments can extend survival, but their efficacy is often limited by toxicity and impaired physical function (e.g. Activities of Daily Living, ADL). Physical frailty, a multidimensional syndrome encompassing reduced strength, physical inactivity, and diminished physiological reserve, is a key barrier to receiving second-line treatments and has been associated with reduced treatment tolerance and shorter survival in patients with unresectable HCC [9]. These observations underscore the importance of improving or maintaining physical function to preserve access to systemic therapies and optimise survival outcomes.

Improvements in physical function may play a critical role in enhancing treatment tolerance and, by extension, survival in patients with HCC. In-hospital physical therapy has been shown to significantly improve functional independence, with higher Functional Independence Measure (FIM) scores following the intervention [10]. These improvements may potentially contribute to clinical outcomes by mitigating frailty, preserving functional status, and enabling continued access to systemic therapies.

Biological mechanisms provide another avenue for exploring the potential link between physical activity and HCC outcomes. Physical activity is known to exert anti-inflammatory effects, which may counteract the chronic inflammation that drives liver disease progression and carcinogenesis [13]. It also enhances insulin sensitivity and glucose metabolism, mitigating the metabolic perturbations commonly associated with HCC. In addition, physical activity promotes favourable changes in adipokines, reduces systemic oxidative stress, and may influence the tumour microenvironment by enhancing immune surveillance and reducing angiogenesis [14, 15]. These putative mechanisms, while requiring further research, form a compelling biological rationale for investigating physical activity as a tool to improve the outcomes for HCC patients.

This structured narrative review aims to synthesize evidence on physical activity and exercise in HCC across:

- Pre-clinical evidence describing biological mechanisms by which exercise may influence tumour and host pathways;
- Observational evidence exploring the association between physical activity, frailty/sarcopenia, and physical function with clinical outcomes in HCC; and.
- Interventional evidence evaluating feasibility, safety, and preliminary efficacy of exercise or rehabilitation interventions in patients with HCC.

By consolidating findings from these areas, the structured narrative review aims to provide a scope of current knowledge and to identify key gaps to inform future translational research.

## Methods

### Design and Reporting Framework

This narrative review was conducted to explore the existing evidence on the potential role of physical activity in improving survival outcomes in patients with HCC. An initial scoping exercise was performed in January 2025 to assess the feasibility of conducting a systematic review. Due to the substantial heterogeneity in study designs, intervention protocols, and outcome measures, a narrative review approach was deemed the most appropriate method for synthesizing the available evidence. Reporting was informed by the PRISMA extension for scoping reviews (PRISMA-ScR), including transparent documentation of information sources and study selection [16].

### Information Sources and Search Strategy

**Fig. 1:**
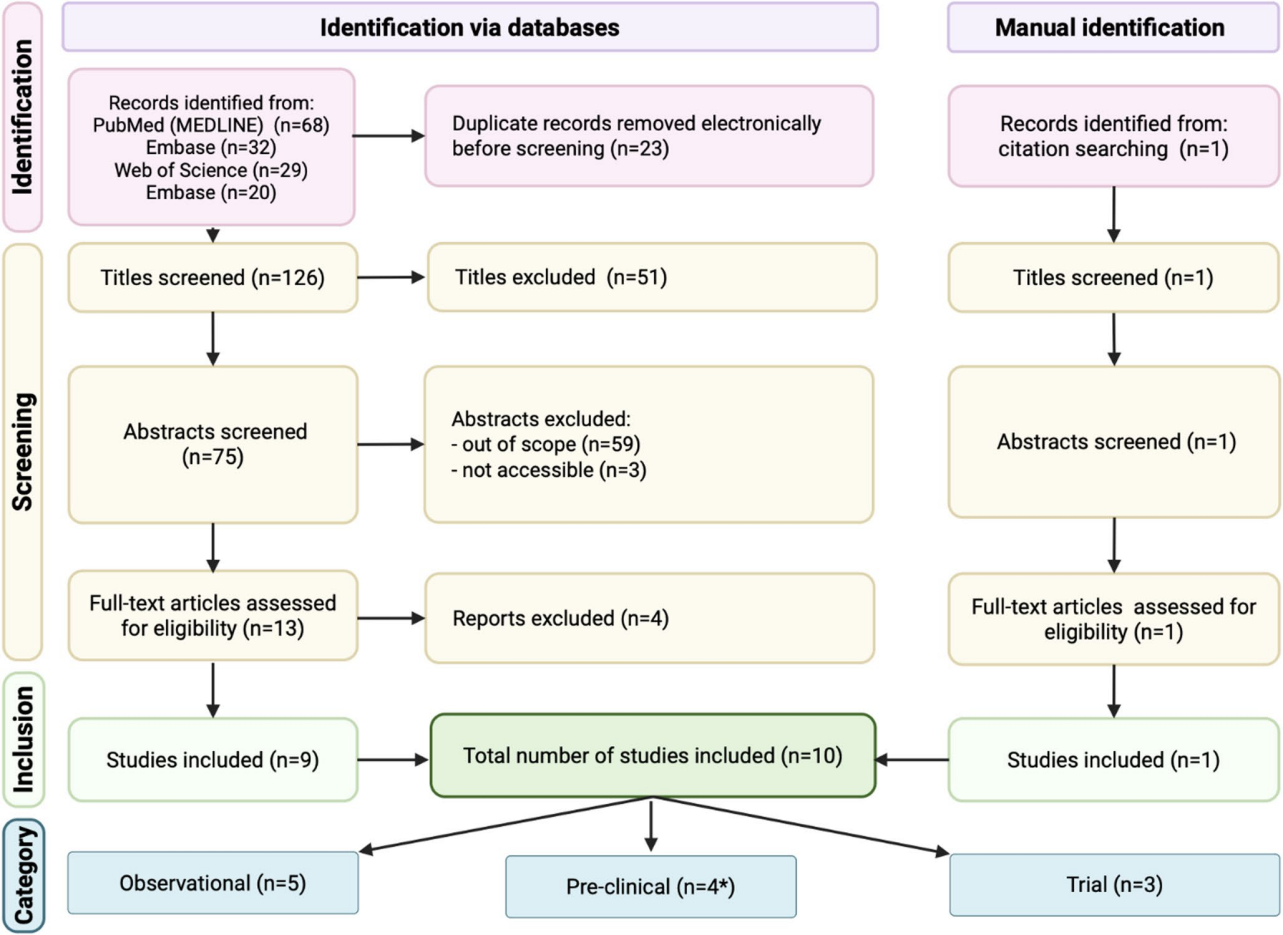
PRISMA 2020 study selection flow diagram. * Liu et al. [20] is categorised into both observational and pre-clinical groups

A literature search was performed on the 21st of March 2025 using four major electronic databases: PubMed (MEDLINE), Scopus, Web of Science, and Embase. Although this is a narrative review, we used the PRISMA 2020 framework to guide transparent reporting of the literature search and study selection process, with the framework described in Fig. 1 [17]. The search strategy combined Medical Subject Headings (MeSH) and free-text terms related to HCC and physical activity. Keywords included combinations of: “hepatocellular carcinoma,” “primary liver cancer,” “physical activity,” “exercise,” “fitness,” “cancer rehabilitation,” “quality of life,” “survivorship,” and “survival.” The search was limited to studies published in English and peer-reviewed journals, with no restriction on publication date. Additional sources were identified through manual searches of key journals, citation tracking of included articles, and examination of reference lists from relevant reviews.

### Eligibility Criteria

Eligibility criteria included original pre-clinical studies describing biological mechanisms by which exercise may influence tumour and host pathways, observational studies exploring the association between physical activity, frailty/sarcopenia, and physical function with clinical outcomes in HCC, and interventional evidence. Studies focusing solely on HCC prevention were excluded. Additionally, qualitative studies that did not specifically examine the impact of physical activity on HCC outcomes were not included.

### Selection of Sources of Evidence

Records were screened in two stages (title/abstract, then full text). Reasons for full-text exclusion were recorded. Common reasons for exclusion included: non-HCC populations, no physical activity/exercise exposure, prevention-only focus without outcomes in HCC, non-primary research, or insufficient outcome reporting. The selection process is summarized in Fig. 1.

### Data Charting and Synthesis

For each included study, we extracted information on study design, population characteristics, aetiology/treatment context where reported, definition and measurement of physical activity/exercise/physical function, outcomes, and key findings. For interventional studies, we extracted intervention characteristics using FITT principles (frequency, intensity, time, type) where available; for observational studies we extracted exposure definitions and measurement methods (e.g., self-report, rehabilitation participation, device-measured activity). The findings were synthesized narratively and organized by evidence category.

### Definitions

*Physical activity* refers to any bodily movement that increases energy expenditure (often measured as an exposure in observational studies). *Exercise* refers to planned, structured, and repetitive physical activity undertaken to improve or maintain fitness. To support consistent reporting of physical activity related variables across studies, the FITT (Frequency, Intensity, Time, Type) framework was used to extract and summarise key characteristics of each intervention [18]. Where available, exercise prescription parameters were tabulated to enable structured comparison between observational, trial, and pre-clinical models. *Physical function* refers to objectively measured performance (e.g., walk tests, sit-to-stand), while frailty reflects reduced physiologic reserve and vulnerability to stressors, and sarcopenia refers to low muscle mass and/or strength. We use these terms consistently throughout.

## Results

### Pre-clinical Mechanisms: Biological Rationale for Exercise in HCC

Pre-clinical studies provide compelling mechanistic insights into how physical activity, specifically aerobic exercise, may directly modulate tumour biology and enhance therapeutic outcomes in HCC (see Table 1). These insights are discussed to address Aim 1.

### Immune Modulation and Tumour Surveillance

Saran and colleagues investigated the effects of aerobic exercise in a syngeneic orthotopic rat model of HCC using Morris Hepatoma-3924 A cells [19]. Rats subjected to daily treadmill running (60 min/day, 5 days/week for 4 weeks) exhibited a marked reduction in tumour viability, cell proliferation, and vascular density compared to sedentary controls. Aerobic exercise increased expression of tumour suppressor PTEN and activated AMP-activated protein kinase (AMPK), while reducing phosphorylation of AKT, S6 ribosomal protein, and STAT3—pathways associated with tumour growth and survival. Interestingly, transcriptomic data suggested that aerobic exercise exerted broader systemic effects on non-tumoural liver tissue, highlighting its role in improving the hepatic microenvironment. These anti-tumour benefits were preserved in animals treated with sorafenib, and similar effects were replicated with metformin, suggesting that structured aerobic exercise may exert metformin-like effects through metabolic and signalling pathway modulation.

Exercise may also function as an immunologic adjuvant to systemic cancer therapy. In a dual clinical-preclinical study, Liu and colleagues demonstrated that physical activity synergized with combined lenvatinib plus anti–PD-1 therapy in mice with HCC [20]. In mice, the addition of aerobic exercise to combination therapy, facilitated by placing running wheels in cages, suppressed tumour growth and significantly prolonged survival. Mechanistically, aerobic exercise reduced regulatory T cell (Treg) infiltration and inhibited immune checkpoint molecule expression (CTLA4, TIGIT, TIM3), thus reprogramming the tumour microenvironment from immunosuppressive to immunostimulatory. These findings support a paradigm in which aerobic exercise not only enhances systemic fitness—defined as the overall capacity of the body to adapt to and recover from various physiological stressors, including cancer treatment—but also directly facilitates anti-tumour immune responses during immunotherapy.

The immunomodulatory effects of exercise may not be limited to the adaptive immune system. In a foundational study across multiple tumour types, including the diethylnitrosamine (DEN)-induced liver tumour model, Pedersen and colleagues showed that voluntary wheel running in mice led to a > 60% reduction in tumour growth, with exercise-induced mobilization and intertumoral infiltration of natural killer (NK) cells [21]. These effects were mediated through epinephrine-induced β-adrenergic signalling and required interleukin-6 (IL-6), which sensitized NK cells to enter the tumour milieu. Blocking either β-adrenergic receptors or IL-6 abolished the tumour-suppressive effects of exercise. These findings implicate sympathetic nervous system activation and cytokine signalling as essential mediators of exercise-driven immunosurveillance.

### Metabolic Signaling and Inflammation

Further evidence for exercise-induced metabolic reprogramming comes from Zhao and colleagues, who assessed aerobic exercise after orthotopic HCC surgery in mice [22]. Using serum metabolomics, they showed that aerobic exercise improved energy metabolism by enhancing tricarboxylic acid (TCA) cycle intermediates such as succinic and citric acid, while reducing stress-related phospholipid metabolites. These metabolic shifts suggest that aerobic exercise helps normalize tumour-disrupted hepatic energy pathways, mitigate mitochondrial stress, and reduce apoptotic signalling via the mitochondrial pathway. The modulation of both mitochondrial function and systemic metabolism supports the notion that exercise restores metabolic balance in post-surgical HCC and may reduce recurrence risk through enhanced physiological recovery.

### Translation Limitations

Taken together, these studies converge on several putative mechanisms by which aerobic exercise may attenuate HCC progression and enhance therapeutic outcomes. These include: (1) modulation of oncogenic signalling pathways such as AKT, mTOR, STAT3, and AMPK; (2) metabolic reprogramming that supports energy efficiency and mitigates cellular stress; (3) reversal of immunosuppression through reduction of Tregs and immune checkpoint markers; and (4) activation and redistribution of innate immune cells like NK cells via neuroendocrine pathways. Importantly, these mechanisms are not mutually exclusive and likely act synergistically to improve both local tumour control and systemic resilience.

This pre-clinical evidence reinforces the biologic plausibility of clinical observations linking physical activity to improved survival and treatment response in patients with HCC. These studies provide mechanistic support for the hypothesis that physical activity may modulate tumour growth, metabolic signalling, and immune function in ways that could enhance therapeutic outcomes. However, while the pre-clinical findings are robust within controlled experimental settings, translation to human populations remains limited. Most clinical studies to date are small, observational, and heterogeneous in design, making it difficult to draw firm conclusions about causality or generalisability. Nevertheless, this mechanistic foundation raises the possibility that exercise may be strategically timed or combined with specific therapies—such as tyrosine kinase inhibitors or immune checkpoint blockade—to optimise benefit. Future translational research should aim to clarify dose–response relationships, identify patient subgroups most likely to benefit, and investigate whether biological markers (e.g., AMPK activation, immune cell profiles) may be used to monitor the effects of exercise in clinical settings.

### Observational Evidence: Frailty and Physical Function as Prognostic Indicators for HCC

To address Aim 2, the emerging epidemiological evidence highlighting the role of physical function in improving clinical outcomes among patients with HCC is discussed, particularly in the context of systemic therapy (see Table 2). Frailty has gained attention as a predictor of survival and treatment tolerance in patients with advanced liver disease. In patients with HCC, frailty may serve as both a surrogate marker of underlying vulnerability and a modifiable determinant of outcomes, with physical activity representing a potential intervention modality.

### Frailty and Treatment Tolerance

Evidence for the prognostic value of frailty was investigated in a prospective, multicentre cohort of 102 patients initiating systemic therapy for HCC [9]. Frailty, measured using the Liver Frailty Index (LFI) —a validated composite score based on grip strength, chair stands, and balance testing— was independently associated with mortality. Patients with higher LFI scores (≥ 4.2) had significantly reduced overall survival, with each unit increase in LFI corresponding to a 74% increased risk of death (adjusted hazard ratio 1.74; 95% CI: 1.17–2.59; *p* = 0.006). Notably, this association persisted even after adjustment for liver function (Child–Pugh score) and albumin–bilirubin grade. Importantly, frailty was not associated with higher rates of disease progression or adverse events but was linked to earlier discontinuation of systemic therapy. One-third of frail patients discontinued treatment prematurely due to physical decline—highlighting how poor functional status can limit therapeutic continuity.

### QoL Trajectories and Physical Resilience

Similar findings were reported by You and colleagues in 2024, who analysed quality-of-life (QoL) trajectories in 156 patients with intermediate to advanced HCC receiving immunotherapy [23]. Using the Functional Assessment of Cancer Therapy-Hepatobiliary (FACT-Hep) tool, the authors identified three distinct QoL trajectories: excellent (35.3%), poor (43.6%), and deteriorating (21.1%). The deteriorating group experienced a sharp decline in QoL within two months of treatment initiation (mean score fell from 124.79 to 98.67), reaching critically low levels by month six (mean 16.58). Multivariate analysis revealed that membership in the deteriorating QoL trajectory was associated with clinical factors such as diabetes, extrahepatic metastasis, and low body mass index—each of which are independently associated with frailty and reduced physical function. These findings underscore the heterogeneity in patient experiences during immunotherapy and suggest that baseline physical resilience, defined as a patient’s intrinsic capacity to withstand and recover from physical stressors (such as illness, treatment side effects, and disease progression), may influence patients’ ability to tolerate and benefit from systemic treatment.

### Rehabilitation, Muscle Mass, and Survival

The benefits of maintaining muscle mass may extend to survival. In a prospective observational study, Hashida and colleagues assessed 152 patients with HCC undergoing TACE, comparing outcomes between those who participated in a structured cancer rehabilitation programme and those who did not [12]. The cancer rehabilitation group experienced greater gains in SMI and had significantly prolonged median survival (552 vs. 424 days; *p* = 0.0359). After propensity score matching, the survival advantage remained (529 vs. 369 days; *p* = 0.0332). In multivariate analysis, cancer rehabilitation participation (estimate 1.760; 95% CI 0.914–3.226; *p* = 0.001) and Child-Pugh class A status were independent predictors of survival. These findings suggest that structured physical rehabilitation may directly influence prognosis in HCC by preserving physical function.

### Exercise and Frailty Improvement

A multicentre observational study by Tsuchihashi and colleagues examined whether structured in-hospital exercise could reduce frailty in patients with HCC [24]. Among 181 hospitalised patients, those in the exercise group (*n* = 114) received a combination of aerobic and resistance training for 20–40 min per day over a median of four days. Improvements in frailty, measured using the Liver Frailty Index (LFI), were significantly greater in the exercise group compared to the non-exercise group (ΔLFI − 0.17 vs. −0.02, *p* = 0.0119). In multivariate analysis, exercise participation was independently associated with frailty improvement (OR 2.38; 95% CI 1.240–4.570; *p* = 0.0091), and decision-tree analysis identified exercise as the primary classifier of LFI change. While these findings suggest that even short-term exercise interventions may help reduce frailty and preserve treatment eligibility, the study’s non-randomised design represents an important limitation. Without random allocation, the potential for selection bias and residual confounding cannot be excluded—particularly given that patients able to participate in exercise may differ systematically from those who did not. As such, although the results are encouraging, they should be interpreted cautiously, and further confirmation in randomised, controlled settings is warranted.

### Physical Activity and Therapy Outcomes

Perhaps the most compelling evidence linking physical activity to therapeutic efficacy comes from Liu and colleagues, who conducted a mixed clinical and pre-clinical investigation into the effects of physical activity in patients receiving combination lenvatinib plus anti–PD-1 therapy for unresectable HCC [20]. In their clinical cohort of 59 patients, those who reported regular physical activity had markedly better outcomes. Compared to sedentary counterparts, physically active patients showed significantly longer overall survival (HR = 0.220, 95% CI: 0.060–0.799), improved progression-free survival (HR = 0.158, 95% CI: 0.044–0.562), and a higher objective response rate (OR = 4.571, 95% CI: 1.482–14.102). These associations remained significant after multivariable adjustment.

Together, these studies provide indicative evidence that frailty status and patient-reported physical function may influence survival, treatment persistence, and wellbeing in patients with HCC. While the available data suggest that reduced physical function is linked to poorer outcomes, the current evidence base is limited by small sample sizes and observational and cross-sectional study designs. Given that frailty is often driven by physical inactivity and deconditioning, structured exercise interventions may represent a pragmatic strategy for mitigating risk, though larger randomised controlled trials (RCTs) are needed to confirm their impact on clinical outcomes including prognosis.

### Interventional Evidence: Exercise as a Feasible and Safe Adjunct

Finally, to address the Aim 3, physical activity is discussed as an increasingly recognised modifiable factor influencing outcomes in patients with HCC. This is particularly relevant given the high prevalence of sarcopenia in this population (around 42%), which is independently associated with reduced survival and limited access to second-line therapies [25]. While traditional HCC management focuses on tumour control and liver function, a growing body of evidence suggests that targeting physical function through structured exercise interventions may improve quality of life, treatment tolerance, and potentially survival (see Table 3).

This is consistent with earlier findings by Koya and colleagues, who evaluated 209 HCC patients undergoing TACE [26]. Those who engaged in moderate-intensity, daily in-hospital exercise showed an increase in skeletal muscle index (SMI), with a mean ΔSMI of + 0.28 cm²/m² compared to − 1.11 cm²/m² in controls (*p* = 0.0029). Exercise was a significant independent predictor of muscle gain (HR 2.13; 95% CI 1.215–3.846; *p* = 0.0085), and 53% of the exercise group achieved muscle hypertrophy compared to 36% of the control group. This reinforces the role of inpatient exercise not only in mitigating treatment-related muscle loss but also in reversing early signs of sarcopenia.

Building on these in-hospital and outpatient models, recent studies have explored the feasibility of delivering exercise remotely, particularly to older patients with comorbidities or limited mobility. The TELEX study by Hallsworth and colleagues evaluated a 10-week telehealth exercise programme in 19 older adults (mean age 74) with HCC who had completed treatment and achieved disease stability [11]. Participants attended supervised, online group exercise sessions twice weekly. Adherence was high (76%), and significant improvements were observed in grip strength (95% CI 1.0 to 5.6), five-times-sit-to-stand performance (95% CI − 3.2 to − 1.2 s), and LFI (95% CI − 0.46 to − 0.23). Patients also reported reduced cancer-related concerns (95% CI 0.30 to 5.85), and no adverse events occurred. Qualitative data underscored the motivational value of real-time instruction and peer interaction, as well as the convenience of exercising from home—highlighting the potential for telehealth to expand access to safe and effective exercise support.

Further supporting the role of remote interventions, Kim and colleagues investigated a 12-week individualised exercise programme delivered via a mobile health (mHealth) platform [27]. Thirty-seven HCC patients received personalised rehabilitation prescriptions and used wearable wristbands to monitor daily activity. Of the 37 enrolled, 84% completed the intervention. Significant improvements were observed in grip strength, the 30-second chair stand test, and the 6-minute walk test from baseline to both 6 and 12 weeks. Additionally, muscle mass and physical activity levels (as measured by IPAQ-SF) increased, all without any biochemical deterioration or hepatic decompensation. The mHealth-supported model offers a scalable, safe alternative to traditional in-person rehabilitation, especially for patients with mobility, geographical, or psychosocial barriers to participation. Although statistically significant improvements were reported in functional outcomes, interpretation of clinical meaningfulness is limited by the absence of established minimal clinically important difference (MCID) thresholds specifically validated in HCC populations. MCID estimates for common functional measures vary across chronic disease and oncology cohorts and depend on baseline impairment and intervention context [28–31]. Accordingly, we interpret these findings as encouraging signals for potential clinical relevance that require confirmation in adequately powered trials with pre-specified MCID thresholds, patient-centred anchors, and longer follow-up.

Taken together, the available studies provide indicative evidence that physical activity interventions may improve physical function, mitigate frailty, preserve muscle mass, and potentially support survival in patients with HCC. This possibility is especially relevant in a clinical context where physical function often determines eligibility for systemic therapies and where frailty is a frequent reason for treatment discontinuation. Notably, across diverse delivery models—including in-hospital, outpatient, telehealth, and mHealth—exercise interventions were generally well tolerated and associated with high adherence, even in older and comorbid populations.

However, the current body of evidence is limited by several key factors. Most studies to date have been observational, underpowered, or designed primarily to assess feasibility rather than efficacy. Sample sizes are typically small, and many interventions lack control groups or randomisation, making results vulnerable to bias and confounding. Inconsistent intervention protocols, outcome measures and follow-up durations further limit the ability to draw firm conclusions about causality or generalisability or to conduct meta-analyses. These limitations highlight the need for well-designed, adequately powered randomised controlled trials to evaluate the clinical effectiveness of exercise interventions in HCC. In parallel, implementation research is needed to develop scalable, integrated models of care that address clinician engagement, referral pathways, and patient education.

## Discussion

### Safety and Clinical Context

Most patients with HCC have underlying cirrhosis and may experience complications that affect exercise feasibility and risk (e.g., ascites, hepatic encephalopathy, sarcopenia, anaemia, thrombocytopenia, falls risk, and variceal bleeding risk) [32–34]. Exercise prescription should therefore be individualised and risk-stratified, particularly because evidence in decompensated subgroups remains limited [35, 36]. In practice, this typically means symptom-guided intensity (e.g., rating of perceived exertion), avoiding high-risk activities in those with balance impairment, close monitoring where ascites or encephalopathy is clinically significant, and coordination with hepatology/oncology teams regarding haemodynamic tolerance and bleeding risk [33, 35, 36]. Safety reporting in the HCC exercise literature is encouraging: in the TELEX-Liver Cancer feasibility study (10-week, virtually supervised group exercise; twice weekly), no adverse events were reported [11]. In the 12-week mobile health (mHealth) intervention (home-based mixed aerobic + resistance), functional improvements were reported without biochemical deterioration or hepatic decompensation [27]. 

### Aetiology and Intervention Heterogeneity

Interpretation is constrained by limited stratification by aetiology (viral hepatitis vs. MASLD vs. alcohol-related liver disease), despite plausible differences in comorbidity patterns, sarcopenia trajectories, and exercise tolerance. Intervention studies also vary substantially in modality (aerobic, resistance, combined), supervision, delivery model (in-hospital vs. home-based vs. telehealth), and outcome selection, complicating dose–response inference and cross-study comparison [32, 37–39]. Future studies should prioritise aetiology-aware analyses and standardised reporting of intervention content using FITT principles, alongside clear documentation of treatment context (e.g., TACE vs. systemic therapy; compensated vs. decompensated cirrhosis) [32, 37].

### Exercise Dose, Modality, and Timing across the Care Pathway

Although HCC-specific FITT parameters are not yet established, the available evidence in this review supports a pragmatic, evidence-aligned prescription anchored to general cancer survivorship guidance and informed by the HCC studies included.

General cancer survivorship recommendations advise building towards at least 150–300 min/week of moderate-intensity aerobic activity (or 75–150 min/week vigorous, or a combination), plus muscle-strengthening activities on at least two days/week [38]. This aligns closely with the “regular physical activity” threshold used by Liu and colleagues in unresectable HCC receiving lenvatinib plus anti–PD-1 therapy: ≥5 days/week of moderate aerobic activity for ≥ 30 min/day (i.e., ≥ 150 min/week), or ≥ 3 days/week of vigorous activity for ≥ 30 min/day, or mixed-intensity patterns meeting comparable criteria [20]. In that cohort, patients meeting this activity definition had substantially improved outcomes (overall survival and progression-free survival) compared with sedentary counterparts [20], supporting the plausibility that achieving at least guideline-level activity may be clinically meaningful in selected patients receiving systemic therapy.

Across interventional studies in this review, combined aerobic and resistance training approaches are most consistently prescribed and clinically reasonable given the high burden of sarcopenia and functional limitation in HCC. In-hospital programmes have used mixed aerobic/resistance training for 20–40 min/day in the short term (median four days) with improvements in frailty indices [24]. Longer programmes delivered remotely or at home have also used mixed-format sessions: TELEX delivered supervised online sessions twice weekly (~ 45 min, moderate perceived exertion) and reported improved strength and frailty metrics without reported adverse events [11], while the mHealth programme encouraged near-daily light–moderate mixed training (~ 40–50 min/session; resistance + walking) with functional gains and no hepatic decompensation/laboratory deterioration [27]. In patients undergoing TACE, daily moderate-intensity inpatient exercise (20–40 min/session) was associated with preservation/improvement of skeletal muscle index in observational comparisons [26].

Given cirrhosis-related symptom burden, physical activity intensity should typically start low-to-moderate and progress based on symptoms and tolerance [35, 36, 38, 39]. Where objective thresholds of monitoring intensity (e.g., based on peak heart rate or metabolic thresholds) are impractical for patients, perceived exertion approaches used in HCC studies (e.g., moderate perceived exertion in TELEX) provide a pragmatic model for scaling intensity safely in clinical settings [11].

Observational evidence in this review indicates that frailty and functional vulnerability are associated with poorer survival and earlier treatment discontinuation during systemic therapy [9], and that quality-of-life trajectories during immunotherapy are heterogeneous, suggesting that baseline resilience may influence tolerance and outcomes [23]. Accordingly, physical activity and functional status assessment may be best positioned at diagnosis, before major treatment transitions (e.g., initiation of systemic therapy; post-locoregional therapy), and during follow-up to detect emerging frailty and trigger timely referral to rehabilitation/exercise support [32, 37].

The reviewed studies suggest that multiple delivery modes may be feasible (inpatient, telehealth, mHealth), which is important given travel burden, comorbidity, and fluctuating symptoms. Implementing this in routine care will likely require multidisciplinary pathways (hepatology/oncology, physiotherapy/exercise physiology, nursing, dietetics) to address sarcopenia risk, symptom burden, safety monitoring, and adherence barriers [38, 39].

### Limitations and Priorities

This evidence base remains limited by small samples, predominance of observational or feasibility designs, heterogeneous exposure and outcome definitions, incomplete aetiology and treatment-context reporting, and substantial risk of confounding and reverse causation (i.e., fitter patients may be more active and also have better outcomes) [32, 37]. Interventional studies are often underpowered for clinical endpoints (e.g., survival, hospitalisation) and adverse events may be under-ascertained or inconsistently reported, limiting confidence in safety estimates across the full spectrum of cirrhosis severity [32, 37]. Finally, although pre-clinical findings provide biological plausibility, they cannot be assumed to translate directly to patients with HCC and cirrhosis [32, 37]. Future work should prioritise prospective, aetiology-aware cohorts with standardised physical activity measurement and pragmatic randomised trials embedded within HCC pathways to establish (i) safety across compensated and decompensated disease, (ii) optimal FITT parameters, and (iii) clinically meaningful endpoints, including treatment tolerance, quality of life, hospitalisation, and survival [32, 37].

## Conclusion

Available evidence suggests that higher physical activity levels and better physical function are associated with more favorable clinical trajectories in HCC, and early interventional studies indicate that exercise and rehabilitation approaches may be feasible and can improve functional outcomes in selected patients. However, the human evidence base remains limited and heterogeneous, and causality cannot be inferred from most existing studies. Pre-clinical data provide biological plausibility but cannot be directly extrapolated to clinical benefit. Future priorities include aetiology-stratified prospective cohorts with standardized physical activity measurement and pragmatic randomised trials embedded within HCC care pathways to establish safety, optimal dose and modality, and clinically meaningful endpoints such as treatment tolerance, hospitalisation, quality of life, and survival.

**Table 1:** Physical activity and hepatocellular outcomes: summary of pre-clinical evidence

Study	Model	Key Findings	FITT
Saran, 2018	Rats with orthotopic Morris Hepatoma-3924 A	Exercise ↓ tumour viability, cell proliferation, and vascularization; ↑ PTEN, pAMPK; ↓ pAKT, pSTAT3, and mTOR signalling. Effects preserved with sorafenib or mimicked by metformin.	F: 5 days/week I: Moderate (motorized treadmill) T: Gradually increasing time T: Aerobic treadmill running
Zhao, 2019	Mice post-orthotopic H22 liver cancer surgery	Aerobic exercise post-surgery ↑ TCA cycle intermediates (e.g., succinate, citrate), ↓ phospholipids and stress metabolites; improved energy metabolism and mitochondrial function.	F: 5 days/week I: 12–18 m/min (moderate–high) T: 60 min/session for 2 weeks T: Aerobic treadmill running
Liu, 2022 (pre-clinical)	Syngeneic mouse model of HCC	Exercise + Lenvatinib/anti-PD-1 ↑ OS and ↓ tumour size; ↓ Treg infiltration, CTLA-4, TIGIT, TIM3 expression; reprogrammed TME from immunosuppressive to immunostimulatory.	F: 5 days/week I: Moderate (voluntary wheel running) T: Approx. 6 weeks T: Voluntary aerobic exercise
Pedersen, 2016	5 mouse tumour models (including DEN-induced liver model)	Exercise ↑ NK cell infiltration and anti-tumour activity via β-adrenergic signalling and IL-6; blocking these pathways abolished tumour suppression effects.	F: Daily (spontaneous) I: Voluntary (varied by mouse) T: Approx. 4–6 weeks T: Voluntary wheel running

↑: upregulatory effect, ↓ : downregulatory effect, DEN: diethylnitrosamine, HCC: Hepatocellular carcinoma, PTEN: Phosphatase and tensin homolog, pAMPK: Phosphorylated AMP-activated protein kinase, pAKT: Phosphorylated protein kinase B, pSTAT3: Phosphorylated signal transducer and activator of transcription 3, mTOR: Mechanistic target of rapamycin, TCA: Tricarboxylic acid, Treg: Regulatory T cells, CTLA-4: Cytotoxic T-lymphocyte-associated protein 4, TIGIT: T cell immunoreceptor with Ig and ITIM domains, TIM3: T-cell immunoglobulin and mucin-domain containing-3, TME: Tumour microenvironment, OS: Overall survival, NK cells: Natural killer cells, IL-6: Interleukin 6

**Table 2:** Physical activity and hepatocellular outcomes: summary of observational evidence

Study	Design	Population	Key Findings
Waller, 2024	Prospective observational study	102 HCC patients starting systemic therapy; LFI assessed	Higher LFI independently predicted death (aHR 1.74; 95% CI: 1.17–2.59); frailty associated with early treatment discontinuation but not with disease progression or AEs.
You, 2024	Trajectory analysis	156 patients with intermediate/advanced HCC on immunotherapy	21.1% experienced severe QoL deterioration (FACT-Hep score ↓ from 124.8 to 16.6); linked with diabetes, metastasis, BMI ≤ 24; poor baseline physical status contributed to decline.
Hashida, 2020	Prospective observational study	152 HCC patients undergoing TACE (CR *n* = 85, control *n* = 67)	CR group had ↑ SMI and ↑ survival (552 vs. 424 days, *p* = 0.0359); survival advantage remained after matching (529 vs. 369 days, *p* = 0.0332).
Liu, 2022	Retrospective study	59 patients with unresectable HCC on Lenvatinib + anti-PD-1	PA group had ↑ OS (HR = 0.220), ↑ PFS (HR = 0.158), ↑ ORR (OR = 4.571); effects remained after adjustment.
Tsuchihashi, 2021	Multicentre observational study	181 HCC inpatients (exercise *n* = 114, non-exercise *n* = 67)	In-hospital exercise significantly improved frailty (ΔLFI − 0.17 vs. -0.02, *p* = 0.0119); exercise independently predicted LFI improvement (OR 2.38; 95% CI: 1.24–4.57).

↑: up regulatory effect, ↓ : downregulatory effect, HCC: Hepatocellular carcinoma, LFI: Liver Frailty Index, OS: Overall survival, aHR: Adjusted hazard ratio, CI: Confidence interval, QoL: Quality of life, FACT-Hep: Functional Assessment of Cancer Therapy–Hepatobiliary, BMI: Body mass index, RT: Radiotherapy, LEN: Lenvatinib, PD-1: Programmed cell death protein 1, PA: Physical activity, PFS: Progression-free survival, ORR: Objective response rate, OR: Odds ratio

**Table 3:** Physical activity and hepatocellular outcomes: summary of interventional evidence

Study	Design	Population	Key Findings	FITT
Koya, 2019	A retrospective case–control study	209 HCC patients undergoing TACE	Exercise group had ↑ SMI (ΔSMI = + 0.28 vs. − 1.11 cm²/m², *p* = 0.0029); exercise was independent predictor of muscle gain (HR 2.13; 95% CI: 1.215–3.846).	F: Daily I: Moderate (2.5 METs) T: 20–40 min/session T: Stretching, strength, balance, endurance (e.g. cycling/walking)
Hallsworth, 2024	Non-randomised feasibility study	19 older adults with stable HCC	10-week online PA improved grip strength, sit-to-stand time, LFI; no AEs; patients reported reduced cancer-related concerns and preferred online delivery.	F: 2x/week I: RPE guided (moderate perceived exertion) T: ~45 min/session T: Warm-up, aerobic/resistance (circuit), cool-down (Zoom-based)
Kim, 2020	mHealth intervention study	37 HCC patients	12-week app-based PA ↑ grip strength, 30s chair stand, 6-min walk; ↑ PA level and muscle mass; no hepatic decompensation or lab deterioration.	F: Daily encouraged I: Light–moderate (specialist-guided, app-monitored) T: 40–50 min/session (20 min resistance + 20–30 min aerobic) T: Home-based mixed PA (resistance + walking)

↑: upregulatory effect, ↓ : downregulatory effect, HCC: Hepatocellular carcinoma, LFI: Liver Frailty Index, ΔLFI: Change in Liver Frailty Index, OR: Odds ratio, CI: Confidence interval, TACE: Transarterial chemoembolization, SMI: Skeletal muscle index, ΔSMI: Change in skeletal muscle index, HR: Hazard ratio, CR: Cancer rehabilitation, PA: Physical activity, 5xSTS: Five-times sit-to-stand test, 6MWT: 6-minute walk test, 30s-CST: 30-second chair stand test, IPAQ-SF: International Physical Activity Questionnaire – Short Form, mHealth: Mobile health

## Data Availability

No datasets were generated or analysed during the current study.

## Abbreviations

ADLs: Activities of Daily Living
AMPK: AMP-Activated Protein Kinase
BCLC: Barcelona Clinic Liver Cancer
CI: Confidence Interval
CR: Cancer Rehabilitation
CTLA4: Cytotoxic T-Lymphocyte–Associated Protein 4
ECOG: Eastern Cooperative Oncology Group
FACT-Hep: Functional Assessment of Cancer Therapy–Hepatobiliary
FIM: Functional Independence Measure
HCC: Hepatocellular Carcinoma
HR: Hazard Ratio
IL-6: Interleukin 6
IPAQ-SF: International Physical Activity Questionnaire–Short Form
IQR: Interquartile Range
LFI: Liver Frailty Index
mHealth: Mobile Health
MASLD: Metabolic Dysfunction–Associated Steatotic Liver Disease
mTOR: Mechanistic Target of Rapamycin
NK cells: Natural Killer Cells
OR: Odds Ratio
ORR: Objective Response Rate
OS: Overall Survival
PFS: Progression-Free Survival
PTEN: Phosphatase and Tensin Homolog
QoL: Quality of Life
STAT3: Signal Transducer and Activator of Transcription 3
SMI: Skeletal Muscle Index
TACE: Transarterial Chemoembolization
TCA cycle: Tricarboxylic Acid Cycle
TIGIT: T Cell Immunoreceptor with Ig and ITIM Domains
TIM3: T-cell Immunoglobulin and Mucin-Domain Containing-3
Tregs: Regulatory T Cells
VEGF: Vascular Endothelial Growth Factor
